# Sinomenine ameliorates cardiac hypertrophy by activating Nrf2/ARE signaling pathway

**DOI:** 10.1080/21655979.2021.2000195

**Published:** 2021-12-13

**Authors:** ManLi Yuan, Bei Zhao, Huaping Jia, Can Zhang, Xiaowen Zuo

**Affiliations:** a Department of Ultrasound Medicine, PLA Strategic Support Force Characteristic Medical Center, Beijing, People's Republic of China; b Department of Cardiovascular Medicine, PLA Strategic Support Force Characteristic Medical Center, Beijing, People's Republic of China

**Keywords:** Oxidative stress, sinomenine, cardiac hypertrophy, apoptosis, Nrf2/ARE

## Abstract

Cardiac hypertrophy (CH) is a result of the physiological adaptation of the heart to coronary heart disease, hypertension, and other cardiovascular diseases. Sinomenine is extracted from *Caulis Sinomenii*. This study aimed to explore the specific mechanism of the action of sinomenine in cardiac hypertrophy (CH) via Nrf2/ARE signaling pathway *in vivo* and *in vitro*. To establish a model of CH, H9C2 cells were treated with angiotensin II (Ang II) and intraperitoneally injected with isoproterenol. Then the cells were treated with 50 and 100 μM sinomenine. TUNEL, HE, rhodamine-labeled phalloidin, and immunohistochemical staining were performed. Flow cytometry was used to measure apoptosis rates. mRNA expression of ANP, BNP, and β-MHC was determined by qRT-PCR. Furthermore, western blotting was performed to analyze protein expression. After sinomenine treatment, the surface area and apoptosis rates were decreased. Furthermore, the mRNA expression of ANP, BNP, and β-MHC, levels of reactive oxygen species and malondialdehyde, and protein expression of Caspase3 and Bax were down-regulated, and the protein expression of Bcl-2 was upregulated. Sinomenine activates the Nrf2/ARE signaling pathway, and inhibition of this signaling pathway reversed the effects of sinomenine. In animal experiments, sinomenine decreased the heart weight and left ventricular weight indices, as well as the expression of ANP, BNP, and β-MHC. Furthermore, sinomenine reduced the apoptosis rate and relieved CH-related oxidative stress by activating the Nrf2/ARE signaling pathway. Together, these findings reveal that sinomenine is a potential candidate drug for CH treatment and further research is required to generalize the result in human subjects.

## Introduction

Cardiac hypertrophy (CH) is a result of the physiological adaptation of the heart to coronary heart disease, hypertension, and other cardiovascular diseases; it mainly manifests as an increase in myocardial cell volume and protein synthesis [1]. CH is also a compensatory response of the heart to maintain proper systolic function and mainly occurs in cases of cardiac overload and injury [2]. When the heart is damaged or overloaded, sympathetic nerve activity is enhanced and circulating catecholamine levels are increased [3], leading to induction or aggravation of ventricular remodeling along with the increased secretion of renin and activation of the renin-angiotensin-aldosterone system in cardiac tissues [4]. Additionally, the increased sympathetic nerve excitability eventually leads to excessive neuroendocrine activation and further aggravates ventricular remodeling [5]. The incidence of CH is high in patients with long-term hypertension and coronary heart disease. Moreover, CH regression can reduce the risk of cardiovascular diseases [6]. However, the pathogenesis of CH is complex, and as there are no specific therapeutic drugs for CH, the identification of effective candidate drugs for CH therapy is of great clinical value.

Currently, research on effective therapeutic approaches for CH is increasingly popular globally [7,8]. However, the specific mechanism underlying the progression of CH is complex and has not been fully elucidated. Many reports have confirmed that oxidative stress is involved in CH pathogenesis. Under CH conditions, lipid peroxidation occurs in cardiomyocytes, affecting their normal function. Oxidative stress contributes to the excessive production of reactive oxygen species (ROS), which activate various hypertrophy signal kinases and transcription factors and lead to pathological remodeling, apoptosis, and contractile dysfunction. Therefore, improving the antioxidant function of cells and restoring the redox balance are important protective mechanisms in the treatment of CH. Increasing evidence has shown that the Nrf2/ARE signaling pathway participates in the initiation of CH and the transition of CH from a compensatory state to heart failure [9,10]. Nrf2/ARE is a classic antioxidant signaling pathway that can remove harmful substances, such as ROS, by regulating the activity of antioxidant enzymes and phase II detoxification enzymes [11]. In addition, the Nrf2/ARE signaling pathway was found to be closely related to cell apoptosis [12] and its activation relieved oxidative damage and apoptosis in cardiomyocytes [13].

In recent years, traditional Chinese medicine has attracted much attention because of its low toxicity and high efficacy; many traditional Chinese medicines have been proven to exhibit preventive and therapeutic effects on CH, such as curcumin [14] and Ginsenoside [15]. Sinomenine (C_19_H_23_NO_4_) is a morphine alkaloid extracted from *Caulis Sinomenii* [16], which exhibits various pharmacological effects, including analgesic, sedative, anti-arrhythmic, anti-oxidant, and anti-inflammatory effects [17,18]. Sinomenine is widely applied in the treatment of rheumatism, arthritis, cancer, and mesangial proliferative nephritis without any obvious side effects [19–21] and has been reported to improve lipopolysaccharide-induced inflammatory injury by inhibiting damage-associated molecular patterns [22]. Previous studies have demonstrated that sinomenine relieved isoproterenol-induced cardiac hypertrophy in mice and may be a promising drug target to improve heart failure [23,24]. However, the mechanism underlying the effects of sinomenine in CH remains unclear.

Therefore, this study focused on the protective effect of sinomenine on CH for the first time. The aim of current study was to explore the mechanism underlying of sinomenine in CH. We hypothesized that sinomenine may reduce apoptosis and oxidative stress in H9C2 cells by regulating the Nrf2/ARE signaling pathway.

## Materials and methods

### Cell culture and treatment

H9C2 cells were obtained from the cell bank of the Chinese Academy of Sciences (Shanghai, China), cultured in Dulbecco’s minimal essential medium containing 10% FBS and 1% penicillin/streptomycin, and placed in an incubator (5% CO_2_, 37°C). To establish the CH model, H9C2 cells cultured to approximately 70% confluence were treated with 1 µM angiotensin II (Ang II) for 48 h. The study groups were as follows: control (CON), Ang II (Model), Ang II+50 μM sinomenine (L-Sin), Ang II+100 μM sinomenine (H-Sin), and Ang II+100 μM sinomenine+10 μM Nrf2 inhibitor All-trans retinoic acid (ATRA) groups. Cells were cultured for 24 h for the following experiments.

### Determination of cell surface area

Cell surface area was measured according to a previous study [25]. Cells in each group were fixed with 4% paraformaldehyde; thereafter, cells were washed and stained with 0.5 mM rhodamine-labeled phalloidin and 0.5 mM DAPI. Fluorescence microscopy was used to photograph the cells, and cell surface area was analyzed using LAS Software (V4.3).

### Determination of malondialdehyde (MDA) and ROS levels in H9C2 cells

MDA (Catalog number, A003-1-2) and ROS (Catalog number, E004-1-1) levels were detected using the corresponding kits provided by Nanjing Jiangcheng Bioengineering Institute (Nanjing, China) according to the manufacturer’s instructions. The specific operation is described according to previous study [26].

### Flow cytometry assay

The apoptosis rate of H9C2 cells was detected using the Annexin V-FITC kit (FA101-01, TransGen Biotech, Beijing, China) as described by Jung et al [27]. Briefly, cells were seeded in 24-well plates, washed, and re-suspended. Next, 5 μL of Annexin V-FITC and 10 μL of PI were added to the cells. Apoptotic cells were detected using a flow cytometer (BD Biosciences, USA).

## TUNEL staining

TUNEL staining was performed according to a previous study [28]. Cells in each group were washed for 5 min and fixed with 4% paraformaldehyde for 30 min. Thereafter, 10% donkey serum (diluted with 0.3% triton) was added to the fixed cells, and the prepared TUNEL reaction solution (FA201-01, TransGen Biotech, Beijing, China) was added under dark conditions (37°C, 1 h). After washing with PBS, cells were stained with DAPI for 5 min. Finally, after washing with PBS, the cells were observed by fluorescence microscopy.

## Quantitative reverse-transcription polymerase chain reaction (qRT-PCR)

mRNA levels of ANP, BNP, and β-MHC were determined using qRT-PCR with the PrimeScript™ RT-PCR Kit (Q111-02/03, Vazyme Biotech Co. Ltd, Nanjing, China). TRIzol (Invitrogen) was used to isolate RNA, and RNA concentration was analyzed using a Nanodrop 2000 spectrophotometer (Mettler Toledo International Trade Co. Ltd, Shanghai, China). M-MLV was used to synthesize cDNA through reverse transcription. For cDNA synthesis, samples were incubated at 43°C for 30 min, 97°C for 5 min, and 5°C for 5 min. The thermal cycling parameters were as follows: 95°C for 5 min and 40 cycles of 95°C for 30 s, 59°C for 30 s, and 72°C for 30 s. GAPDH was used as an internal control. Relative gene expression was calculated using the 2^−ΔΔCt^ method according to the previous study [29]. The primer sequences are as follows:

ANP, Forward: 5ʹ-GACGCCCTCCGATGTGAAAG-3ʹ, Reverse: 5ʹ-GGCTCTGTTACTGCTTAGTTCAA-3ʹ;

BNP, Forward: 5ʹ-CAGAAGGTGCTGCCCCAGATG-3ʹ, Reverse: 5ʹ-GACTGCGCCGATCCGGTC-3ʹ;

β-MHC, Forward: 5ʹ-TTTGATGTGCTGGGCTTCAC-3ʹ, Reverse: 5ʹ-TGACATACTCGTTGCCCACT-3ʹ.

## Western blot

Western blot assay was conducted according to a previous study [30]. Cell protein was collected using RIPA lysis buffer (Thermo Fisher Technology Co. Ltd, China), and protein concentrations were measured using a BCA kit (Thermo Fisher Technology Co. Ltd). Proteins (35 μL) were electrochemically transferred to a PVDF membrane by polyacrylamide gel electrophoresis. After blocking the membrane with 5% skim milk for 2 h, the membrane was treated with anti-Caspase3 (ab32351, 1:600, Abcam, USA), anti-Bax (ab32503, 1:1000, Abcam), anti-Bcl2 (ab32124, 1:800, Abcam), anti-Nrf2 (ab62352, 1:1000, Abcam), anti-HO-1 (ab52947, 1:1000, Abcam), and anti-GAPDH (ab8245, 1:500, Abcam) overnight at 4°C. Next, the membrane was treated with horseradish peroxidase-conjugated secondary antibodies (ab150077, 1: 2000, Abcam) for 2 h. Finally, after developing with ECL at room temperature, the gray value was analyzed using ImageLab software. GAPDH was used as an internal reference.

## Animal experiments

The animal experiments were approved by the ethical committee of the Strategic Support Force Medical Center (2,020,092,023). The mice were obtained from Charles River Laboratories (Beijing, China). Forty-eight healthy mice (24 male and 24 female) in the C57BL/6 background were randomly divided into a control group (CON), 10 mg/kg isoproterenol (ISO) model group (Model), ISO + 40 mg/kg sinomenine group (L-Sin), and ISO + 80 mg/kg sinomenine group (H-Sin). Except for mice in the CON group, mice in the other groups were intraperitoneally injected with isoproterenol for 14 days. The CON group received the same amount of normal saline daily. Four hours after the injection of ISO every morning, sinomenine was administered by gavage once daily. The CON and Model groups were administered the same amount of normal saline by gavage for 4 weeks.

## Determination of cardiac mass index

The body weights (BW) of the mice were measured at 12 h after the last administration. The heart was collected and washed with normal saline solution. Heart weights (HWs) and the left ventricular weights (LVWs) were measured, and the HW index (HWI = HW/BW) and LVW index (LVWI = LVW/BW) were calculated as described by Dludla et al. [31].

## Hematoxylin and eosin (HE) staining

The HE staining was performed according to a previous study [32]. The left ventricle was collected, fixed with 4% paraformaldehyde, taken transversely (0.5 cm^3^), embedded in paraffin, and sectioned (5 μm). HE staining was used to observe the morphological changes in the myocardium under a light microscope.

## Immunohistochemical staining

The immunohistochemical staining was conducted as described by Qu et al. [33]. The tissue sections were treated with 1% Triton-100 for permeabilization after dewaxing and rehydration and were treated with 3% H_2_O_2_ for 15 min. The sections were blocked with 5% goat serum and treated with anti-ANP, anti-BMP, and anti-β-MHC antibodies overnight at 4°C. The next day, the corresponding secondary antibody (37°C, 30 min) was added. The sections were incubated in DAB substrate for Color reaction. Finally, the sections were stained and observed.

## Statistical analysis

Data are expressed as the mean ± standard deviation (SD). SPSS20.0 software was used for statistical analysis. All data conformed to normal distribution. To compare the differences between groups, one-way analysis of variance (ANOVA) was performed, followed by the Bonferroni post-hoc test. Statistical significance was set at P < 0.05.

## Results

This study focused on the protective effect of sinomenine on CH for the first time. We aimed to explore the mechanism underlying of sinomenine in CH. We demonstrated that sinomenine may reduce apoptosis and oxidative stress in H9C2 cells by regulating the Nrf2/ARE signaling pathway. And sinomenine down-regulated the ANP, BNP and β-MHC levels in CH *in vivo* and *in vitro*.

## Sinomenine decreased surface area and ANP, BNP and β-MHC levels in Ang II-treated H9C2 cells

**Figure 1.**
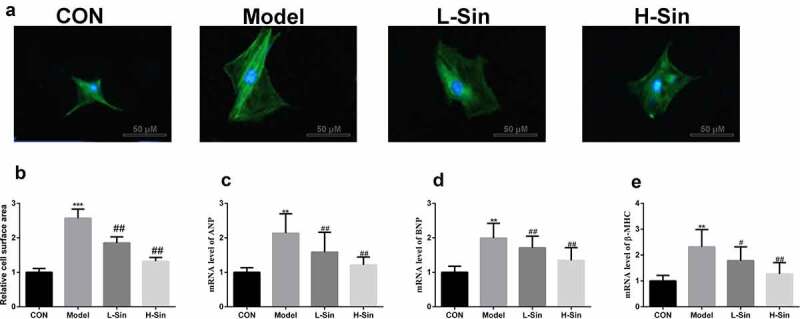
Sinomenine decreased the surface area and the mRNA levels of ANP, BNP and β-MHC of Ang II-treated H9C2 cells H9C2 cells were stained with rhodamine-labeled phalloidin and DAPI. **b** Cell surface area was analyzed using LAS software. **c-e** mRNA levels of ANP, BNP, and β-MHC were detected using qRT-PCR. **p < 0.01, ***p < 0.001 vs. CON. #p < 0.05, ##p < 0.01 vs. Model

To explore the effect of sinomenine on CH, we created a CH cell model with Ang II. Compared with the CON group, the Model group showed a significant increase in the cell surface area (P < 0.05). Sinomenine dramatically decreased the cell surface area in a dose-dependent manner (P < 0.05, Figure 1a-b). Additionally, the mRNA expression of ANP, BNP, and β-MHC was significantly upregulated in the Model group than in the CON group (P < 0.05), while sinomenine dramatically downregulated the ANP, BNP, and β-MHC levels in a dose-dependent manner (P < 0.05, Figure 1c-e).

## Sinomenine reduced the apoptosis rate of Ang II-treated H9C2 cells

**Figure 2.**
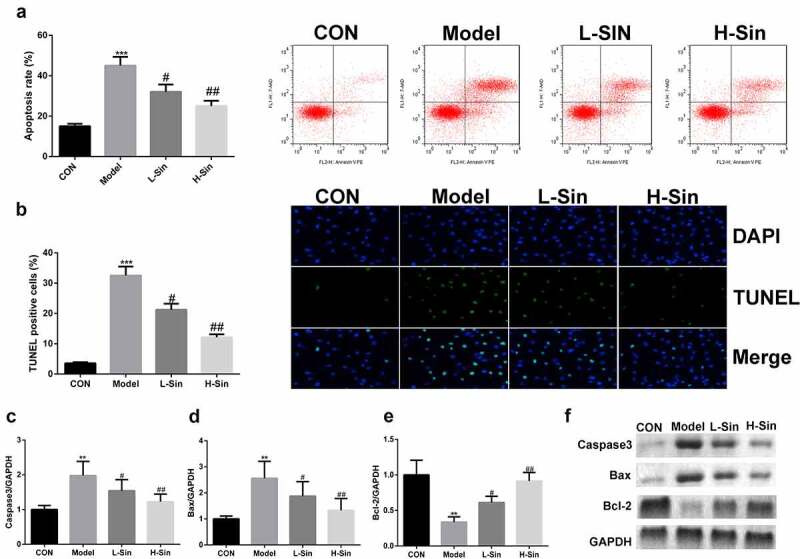
Sinomenine reduced the apoptosis rate of Ang II-treated H9C2 cells Apoptosis rates of H9C2 cells were determined using flow cytometry. **b** TUNEL staining was used to observe apoptosis in H9C2 cells. **c-f** Protein expression of Caspase3, Bax, and Bcl-2 was measured by western blotting. **p < 0.01, ***p < 0.001 vs. CON. #p < 0.05, ##p < 0.01 vs. Model

We further explored the effect of sinomenine on the apoptosis rate of Ang II-treated H9C2 cells. The apoptosis rate was significantly higher in the model group than in the CON group, and sinomenine dramatically decreased the apoptosis rate in a dose-dependent manner (P < 0.05, Figure 2a), which was consistent with the results of TUNEL staining (Figure 2b). Furthermore, western blotting was used to detect the protein expression of Caspase3, Bax, and Bcl-2; compared to the CON group, the Model group showed upregulated expression of Caspase3 and Bax downregulated expression of Bcl-2 (P < 0.05). Sinomenine dramatically downregulated the expression of Caspase3 and Bax and upregulated the expression of Bcl-2 in a dose-dependent manner (P < 0.05, Figure 2c-f).

## Sinomenine relieved oxidative stress through activation of the Nrf2/ARE signaling pathway

**Figure 3.**
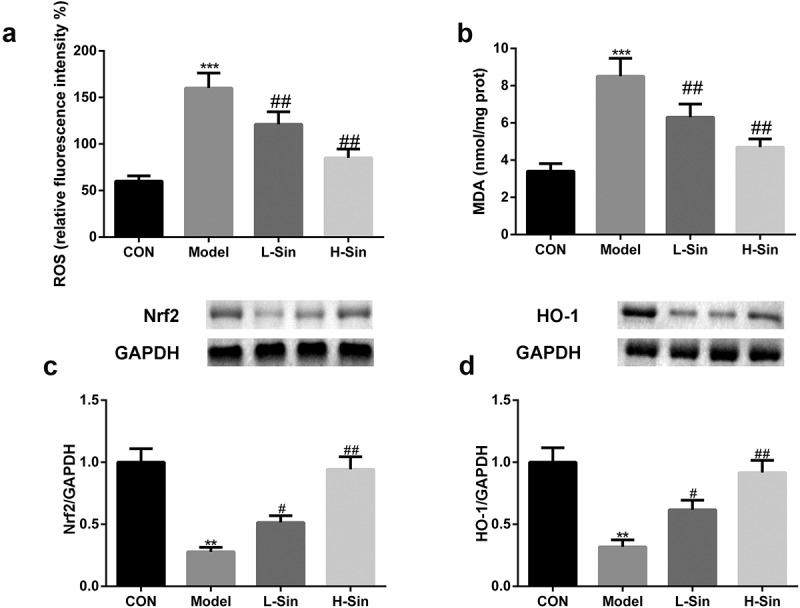
Sinomenine relieved oxidative stress through activation of the Nrf2/ARE signaling pathway **a-b** ROS and MDA levels in Ang II-treated H9C2 cells **c-d** Protein expression of Nrf2 and HO-1 was measured by western blotting. **p < 0.01, ***p < 0.001 vs. CON. #p < 0.05, ##p < 0.01 vs. Model

Subsequently, we investigated the effects of sinomenine on the Nrf2/ARE signaling pathway. As shown in Figure 3, MDA and ROS levels were markedly upregulated in the Model group than in the CON group (P < 0.05); sinomenine dramatically decreased these MDA and ROS levels in a dose-dependent manner (P < 0.05, Figure 3a-b). In addition, we found that the protein expression of Nrf2 and HO-1 was significantly decreased in H9C2 cells of the Model group compared with that in H9C2 cells of the CON group (P < 0.05); sinomenine dramatically increased the protein expression of Nrf2 and HO-1 in a dose-dependent manner (P < 0.05, Figure 3c-d).

## ATRA inhibited the Nrf2/ARE signaling pathway

**Figure 4.**
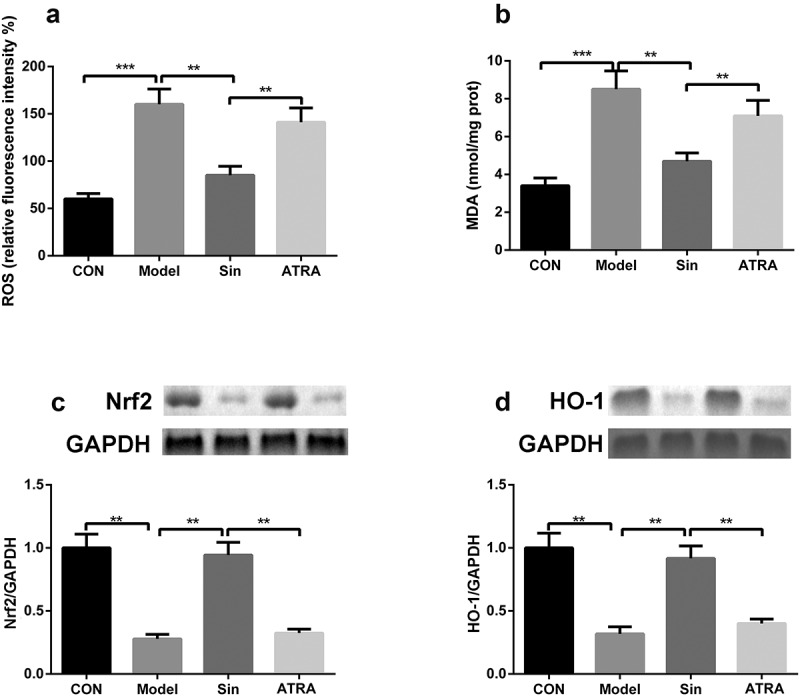
ATRA inhibited the Nrf2/ARE signaling pathway **a-b** ROS and MDA levels in Ang II-treated H9C2 cells after ATRA treatment. **c-d** Protein expression of Nrf2 and HO-1 was measured by western blotting after ATRA treatment. **p < 0.01

To explore the specific mechanism underlying the function of the Nrf2/ARE signaling pathway in CH, H9C2 cells were treated with 100 μM sinomenine and the Nrf2 inhibitor ATRA. ATRA reversed the effects of sinomenine on the levels of MDA and ROS (P < 0.05, Figure 4a-b) and the protein expression of Nrf2 and HO-1 (P < 0.05, Figure 4c-d).

## Inhibition of the Nrf2/ARE signaling pathway increased surface area and ANP, BNP, and β-MHC levels in Ang II-treated H9C2 cells

**Figure 5.**
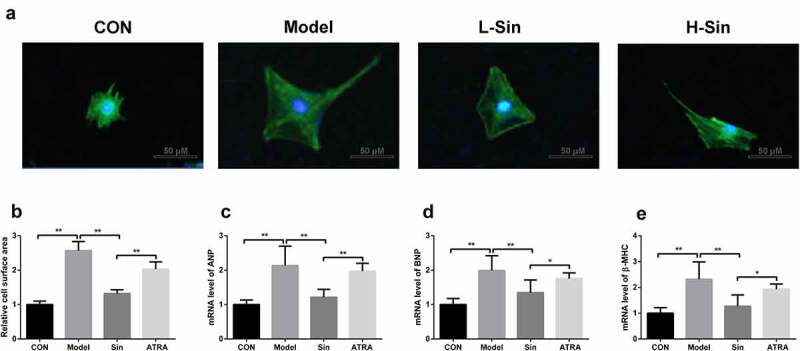
Inhibition of the Nrf2/ARE signaling pathway increased the surface area and the mRNA levels of ANP, BNP and β-MHC of Ang II-treated H9C2 cells H9C2 cells were stained with rhodamine-labeled phalloidin and DAPI. **b** Cell surface area was analyzed using LAS software. **c-e** mRNA levels of ANP, BNP, and β-MHC were detected using qRT-PCR. *p < 0.05, **p < 0.01

Subsequently, we noted that the cell surface area in the ATRA group was significantly higher than that in the Sin group (P < 0.05, Figure 5a-b). In addition, the levels of ANP, BNP, and β-MHC were increased after ATRA treatment compared to those in the Sin group (P < 0.05, Figure 5c-e).

## Inhibition of the Nrf2/ARE signaling pathway increased the apoptosis rate of Ang II-treated H9C2 cells

**Figure 6.**
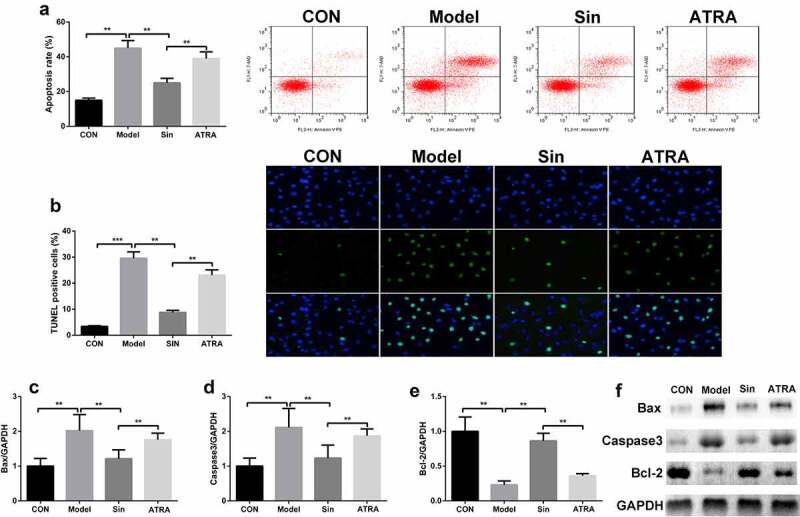
Inhibition of the Nrf2/ARE signaling pathway increased the apoptosis rate of Ang II-treated H9C2 cells Apoptosis rates of H9C2 cells were determined using flow cytometry. **b** TUNEL staining was used to observe apoptosis in H9C2 cells. **c-f** Protein expression of Caspase3, Bax, and Bcl-2 was measured by western blotting. **p < 0.01

Compared with the Sin group, the ATRA group showed an increase in the apoptosis rate of H9C2 cells (P < 0.05, Figure 6a), which was consistent with the results of TUNEL staining (Figure 6b). Furthermore, the protein expression of Caspase3 and Bax was upregulated and that of Bcl-2 was downregulated in the ATRA group compared to those in the Sin group (P < 0.05, Figure 6c-f).

## Sinomenine inhibited ISO-induced CH in mice

**Figure 7.**
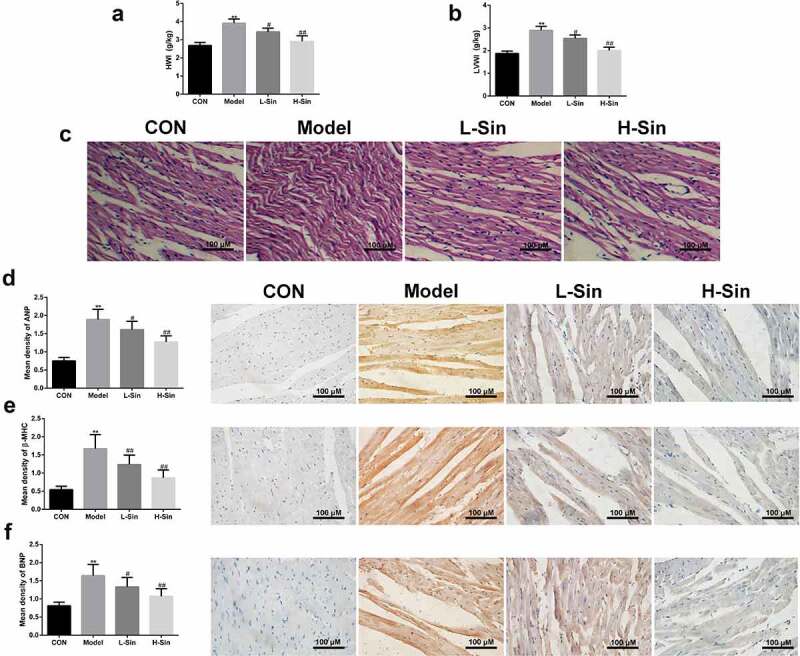
Sinomenine inhibited ISO-induced CH in mice **a-b** Heart weight index (heart weight/body weight; HWI) and left ventricular weight index (left ventricular weight/body weight; LVWI). **c** HE staining was used to observe the morphological structure of the heart tissue. **d-f** Immunohistochemical staining was performed to measure the expression levels of ANP, BNP, and β-MHC. **p < 0.01 vs. CON. #p < 0.05, ##p < 0.01 vs. model

Finally, we verified the effects of sinomenine on ISO-induced CH in mice. As shown in Figure 7, HWI and LVWI were markedly increased in mice of the Model group compared to those in mice of the CON group. Sinomenine treatment significantly decreased the ISO-induced increase in HWI and LVWI in mice (P < 0.05, Figure 7a-b). In in the Model group, HE staining also showed hypertrophy and disordered arrangement in cardiomyocytes as well as an increase in the transverse diameter and surface area of cardiomyocytes (Figure 7c). In addition, consistent with the results of the cellular experiments, immunohistochemical staining also showed that the protein expression of ANP, BNP, and β-MHC in the Model group was significantly increased (P < 0.05), and this increase was significantly decreased by treatment with sinomenine (P < 0.05, Figure 7d-f).

## Discussion

In this study, we confirmed that sinomenine can suppress oxidative stress and apoptosis in Ang II-treated H9C2 cells by regulating the Nrf2/ARE signaling pathway and inhibiting ISO-induced CH in mice. Our findings indicate that sinomenine may be a potential candidate drug for the treatment of CH.

CH is characterized by physiological and pathological cell enlargement [34], and apoptosis has been reported to be closely related to CH [35]. Apoptosis is an important factor in cardiovascular diseases, autoimmune diseases, and other diseases [36]. Sinomenine, an alkaloid monomer, is the main component of *Caulis Sinomenii* and has anti-inflammatory, analgesic, and immunosuppressive effects [18]. However, there are few reports on the protective effects of sinomenine on CH. Gao et al. [37] showed that sinomenine relieves intervertebral disc degeneration by suppressing apoptosis *in vitro* and *in vivo*. Additionally, Fu et al. [38] confirmed that sinomenine protects neuronal cells by suppressing apoptosis. Other studies also suggested that sinomenine relieved isoproterenol-induced cardiac hypertrophy and may be a promising drug target to improve heart failure [22,23]. Correspondingly, this study showed that sinomenine upregulates Bcl-2 expression, downregulates Caspase3 and Bax expression, and decreases the apoptosis rate of Ang II-treated H9C2 cells, indicating that sinomenine may relieve CH development by stimulating the anti-apoptotic effects of Bcl-2 and intercepting the pro-apoptotic effects of Caspase3 and Bax.

CH is the response of a healthy heart to increasing external and internal biomechanical stress, which manifests as an increase in cardiac cell volume [39]. A previous study confirmed that ANP and BNP are markers of CH. ANP is a circulating peptide secreted by the atrium that can be activated during CH development; it is a component of the natriuretic peptide system and is often used as a prognostic marker in patients with compensatory heart failure or CH [40]. Furthermore, overexpression of β-MHC in the heart leads to impaired myocardial function, and the inhibition of β-MHC expression can prevent the occurrence of ISO-induced CH [41]. An increasing amount of evidence has shown that the levels of ANP, BNP, and β-MHC as well as the cell surface area are increased in CH [42,43]. This study confirmed that sinomenine can cause a decrease in the cell surface area and can downregulate the levels of ANP, BNP, and β-MHC *in vitro* and *in vivo*. These results illustrate that sinomenine may be an effective drug for the treatment of CH.

To further investigate the specific mechanism of sinomenine on CH, we verified the role of sinomenine in the Nrf2/ARE signaling pathway. Nrf2 is a key transcription factor in the cell antioxidant system and plays a key role in maintaining intracellular redox homeostasis [44]. Under normal physiological conditions, Nrf2 binds to Keap1 in a degradable and inactive state in the cytoplasm. During oxidative stress, Nrf2 dissociates from Keap1 and translocates to the nucleus, where it binds to nuclear ARE and activates the transcription of downstream antioxidant-related genes [45]. Oxidative stress has been demonstrated to be a key factor in the progression of CH. Nie et al. [46] confirmed that astragaloside suppresses CH development by activating the Nrf2 signaling pathway. Velusamy et al. [47] indicated that oxidative stress is a major factor in CH and highlighted that hesperetin can cure ISO-induced CH by targeting Nrf2. These findings illustrate that Nrf2 is a novel therapeutic target for treating CH. This study also showed that sinomenine can decrease MDA and ROS levels and activate the Nrf2/ARE signaling pathway in Ang II-treated H9C2 cells. Interestingly, this study confirmed that the inhibition of Nrf2 reversed the effects of sinomenine. These results further confirm that the effects of sinomenine on CH through the Nrf2/ARE signaling pathway.

### Conclusion

In conclusion, this study found that sinomenine reduces apoptosis and relieves oxidative stress by regulating the Nrf2/ARE signaling pathway, which was also verified *in vivo*. Our results indicated that sinomenine may be a promising drug target to the treatment of CH. However, there is still some limitation in this study, these results need to be further confirmed clinically and cardiac function of the patients should be measured.

## Highlights

- Sinomenine decreased the ANP, BNP, and β-MHC levels
- Sinomenine relieved the apoptosis and oxidative stress
- Nrf2/ARE signaling pathway was activated by sinomenine
